# ZnS–rGO/CNF Free-Standing Anodes for SIBs: Improved Electrochemical Performance at High C-Rate

**DOI:** 10.3390/nano13071160

**Published:** 2023-03-24

**Authors:** Debora Maria Conti, Cristina Fusaro, Giovanna Bruni, Pietro Galinetto, Benedetta Albini, Chiara Milanese, Vittorio Berbenni, Doretta Capsoni

**Affiliations:** 1 Department of Chemistry, Physical Chemistry Section & C.S.G.I. (Consorzio Interuniversitario per lo Sviluppo dei Sistemi a Grande Interfase), University of Pavia, 27100 Pavia, Italy; deboramaria.conti01@universitadipavia.it (D.M.C.); cristina.fusaro01@universitadipavia.it (C.F.); giovanna.bruni@unipv.it (G.B.); chiara.milanese@unipv.it (C.M.); vittorio.berbenni@unipv.it (V.B.); 2 Department of Physics, University of Pavia, 27100 Pavia, Italy; pietro.galinetto@unipv.it (P.G.); benedetta.albini01@universitadipavia.it (B.A.)

**Keywords:** electrospinning, carbon nanofibers, zinc sulfides anode, sodium-ion batteries, self-standing anodes, binder-free anodes

## Abstract

ZnS–graphene composites (ZnSGO) were synthesized by a hydrothermal process and loaded onto carbon nanofibers (CNFs) by electrospinning (ZnS–GO/CNF), to obtain self-standing anodes for SIBs. The characterization techniques (XRPD, SEM, TEM, EDS, TGA, and Raman spectroscopy) confirm that the ZnS nanocrystals (10 nm) with sphalerite structure covered by the graphene sheets were successfully synthesized. In the ZnS–GO/CNF anodes, the active material is homogeneously dispersed in the CNFs’ matrix and the ordered carbon source mainly resides in the graphene component. Two self-standing ZnS–GO/CNF anodes (active material amount: 11.3 and 24.9 wt%) were electrochemically tested and compared to a tape-casted ZnS–GO example prepared by conventional methods (active material amount: 70 wt%). The results demonstrate improved specific capacity at high C-rate for the free-standing anodes compared to the tape-casted example (69.93 and 92.59 mAh g^−1^ at 5 C for 11.3 and 24.9 wt% free-standing anodes, respectively, vs. 50 mAh g^−1^ for tape-casted). The 24.9 wt% ZnS–GO/CNF anode gives the best cycling performances: we obtained capacities of 255–400 mAh g^−1^ for 200 cycles and coulombic efficiencies ≥ 99% at 0.5 C, and of 80–90 mAh g^−1^ for additional 50 cycles at 5 C. The results suggest that self-standing electrodes with improved electrochemical performances at high C-rates can be prepared by a feasible and simple strategy: ex situ synthesis of the active material and addition to the carbon precursor for electrospinning.

## 1. Introduction

It is an established fact that the world’s energy demand is constantly increasing. The refined fossil fuels commonly used in the past cannot be considered as suitable energy sources for the future, due to environmental pollution and global warming concerns. Based on this, in recent decades researchers have focused growing attention on developing a variety of sustainable, efficient, and renewable energy storage systems [1].

Rechargeable batteries have been regarded as promising candidates in advanced energy storage technology; among them, lithium-ion batteries (LIBs) have demonstrated their suitability and wide applicability as energy storage devices. These have been successfully employed in several small devices (mobile phones, iPads, laptops), thanks to their high energy density and long-cycle life [2,3]. The wide experience and knowledge acquired over the past three decades regarding LIBs make them a state-of-the-art technology, also suitable for applications in hybrid- and plug-in-electric vehicles. However, the limited and uneven distribution of lithium resources on the earth’s crust implies increasing costs [4,5,6], causing shortcomings in large-scale application of LIBs in the emerging markets of electrical vehicles and energy storage systems. Among rechargeable batteries, sodium-ion batteries (SIBs) represent a viable alternative to LIBs: Na is abundant on the earth’s crust, is low-cost, and displays similar chemistry and redox potential, only slightly higher than lithium (−2.71 V vs. SHE and −3.04 V vs. SHE for Na^+^/Na and Li^+^/Li, respectively). Stimulated by these factors, some companies have invested money and research into SIBs, and in some cases these devices are actually at the commercialization and large scale production stage [7]. However, the larger size of the sodium ion (1.02 Å) compared to the lithium ion (0.76 Å) poses concerns about the choice of the cathode and anode materials suitable for sodiation/desodiation processes and for satisfying requisites such as adequate specific capacity, high coulombic efficiency and long life-cycle.

On the anode side, several materials were investigated, such as various carbons [8], alloys [9], transition metal oxides [10] and transition metal sulfides [11]. Carbonaceous materials exhibit high surface area and conductivity, but theoretical capacity and coulombic efficiency are not impressive [12,13]; improvements in terms of fast and stable sodium storage and high performance for SIBs have been obtained very recently by using porous-doped or vanadium-modified carbon materials [14,15]. The alloys’ main concern is the huge volume change during the sodiation/desodiation process, which affects cell stability and cyclability. The transition metal sulfides seem attractive, as they display a high capacity compared to transition metal oxides and relatively reversible Na_2_S kinetics compared to the Na_2_O [16,17]. Among the transition metal sulfides, ZnS is undoubtedly an interesting candidate for SIBs, as it is non-toxic, low cost, and abundant on the earth’s crust. It displays a capacity of about 550 mAh g^−1^ and a redox potential below 0.5 V, desirable for the achievement of high energy density [18]. However, it suffers from huge volume expansion/contraction during the sodiation/desodiation processes and displays poor intrinsic conductivity [19]; moreover, electrode pulverization due to dissolution of polysulfides upon sodiation occurs [20]. All these undesired features hinder the assembly of SIBs with outstanding electrochemical performances. Various approaches have been proposed to solve the above mentioned problems, based on the realization of peculiar morphologies, on the construction of nano- and porous-structures and on the synthesis of ZnS–carbon composites [21,22,23,24,25].

All the above mentioned strategies offer an effective means to buffer the volume change and to enhance ion diffusion and electrolyte wetting of the active material. In fact, high performance electrodes are obtained by optimal ionic and electronic conductivity. The former is achieved when the electrode porosity allows a proper electrolyte permeation. In this way, a fast ion diffusion and ionic contact between active material particles and ions is reached [26]; low electrode porosity diminishes wettability and decreases discharge capacity [27,28]. Indeed, the carbon coating/embedding strategy also plays a relevant role in solving the poor electronic conductivity of ZnS.

Among carbonaceous sources, carbon nanofibers (CNFs) have been demonstrated as appealing materials for application in LIBs and SIBs. Their peculiar quasi-one-dimensional structure provides high surface-to-volume ratio, high porosity, short transport distance for ions and easy permeation of the electrolyte. They possess excellent electronic conductivity, high mechanical strength and good flexibility. All the above mentioned features make CNFs suitable conductive fillers [29], conductive supports for cathodic and anodic materials (self-standing and binder-free electrodes) [30,31,32,33,34], and anodic active material by themselves [35,36,37].

Regarding metal sulfide anodes, recent literature has reported improved electrochemical performances for nanoscale active materials homogeneously embedded into CNFs [38]; several metal sulfides were investigated, such as MoS_2_ [33], CoS_2_ [39], Fe_7_S_8_ [40], Ni_x_S_y_ [41] and Cu_9_S_5_ [42].

As concerns the synthetic strategy, different approaches have been employed to prepare active material–CNF composites, mainly based on the use of the electrospinning technique as a feasible, easy to manipulate, scalable and controllable process. In some cases, the carbon precursor solution is electro-spun, stabilized and carbonized to obtain the CNFs, then a solution containing the active material precursors is dip-/drop-coated on the CNFs, which are finally heat- or chemically-treated to obtain the final product. In other cases, it is considered more convenient to mix the solutions of carbon and the active material precursors: the obtained solution is electro-spun and undergoes chemical and thermal treatments suitable for obtaining the free-standing anode.

As regards ZnS–CNF composites, two very recent papers have been reported in the literature. Wei et al. [23] synthesized bell string-like hollow ZnS–C nanofiber films, by integrating hollow ZnS derived from a metal-organic framework precursor and three-dimensional N, S co-doped carbon nanofiber networks by an electrospinning technique. The obtained film undergoes solvothermal sulphuration and pyrolysis processes. The free-standing anode is tested for SIBs and shows superior rate capabilities (258.3 mA h g^−1^ at 10 A g^−1^ and a high initial coulombic efficiency of 88.4%) and cycling stability after 500 cycles at 1 A g^−1^. Wang et al. [43] synthesized a ZnS nanocrystals-high porosity carbon fibers hybrid material by one-step electrospinning: they used zinc diethyldithiocarbamate and polyacrylonitrile as raw materials and poly (ethylene glycol)–block-poly (propylene glycol)–block-poly (ethylene glycol) as template. In this approach the sulphuration process is avoided. The composite, applied to LIBs, shows a specific capacity of 592.2 mAh g^−1^ under a current density of 1 A g^−1^ after 1000 cycles.

In the present work, we investigate a different approach to preparing ZnS–graphene nanocrystals in carbon nanofibers. We develop a two-step strategy. First, we apply a conventional synthetic route (hydrothermal synthesis) to prepare ZnS–graphene composites. Then, the obtained active material was simply added in the proper amount to the carbon precursor solution and the dispersion was electro-spun. The film was stabilized and carbonized to obtain the self-standing anode. The structure, the morphology, the active material amount and the order degree of the carbonaceous component are investigated by X-Ray powder diffraction, scanning electron microscopy, transmission electron microscopy, thermogravimetric analysis and Raman spectroscopy. The electrochemical performances of the self-standing anodes are tested and compared to the ZnS–graphene anode prepared by conventional tape-casting route. In summary, we demonstrate that a homogeneous distribution of ZnS–graphene in CNFs and improved electrochemical performances at high C-rates can be obtained by a simple synthetic strategy based on adding the active material to the carbon precursor solution and electrospinning the dispersion.

## 2. Materials and Methods

### 2.1. Materials

All the chemicals employed were reagent grade or of higher quality. Graphene oxide (GO), zinc acetate dihydrate (Zn(CH_3_COO)_2_·2H_2_O), ammonium hydroxide solution (NH_4_OH, ≥25% NH_3_ basis), sodium sulfide nonahydrate (Na_2_S·9H_2_O), 1 M sodium perchlorate in EC:DEC (1:1 *v*:*v*) electrolyte, fluoroethylene carbonate (FEC), carbon black Super P powder, carboxymethyl cellulose (CMC), polyacrylonitrile (PAN: (C_3_H_3_N)_n_), and N,N-dimethylacetamide (DMAc: CH_3_CON(CH_3_)_2_) were purchased from Signa-Aldrich (Milano, Italy).

### 2.2. Synthesis

#### 2.2.1. Active Material ZnS–Graphene

ZnS–graphene (ZnS–GO) composite is prepared through hydrothermal synthesis as reported by Zhang et al. [18], with some modifications. The solution containing the reagents is placed inside an autoclave and the reaction is conducted under high pressure and temperature conditions. The obtained product is a hydrogel, which is then dried in air. Hereafter we report the synthesis procedure in detail.

100 mg of graphene oxide were dispersed in 60 mL of distilled water; the suspension was sonicated (Ultrasonic Cleaner, DU-65) to obtain a homogeneous dispersion, then 4 mmol of zinc acetate dihydrate were added and sonicated for 1 h. The suspension was basified at pH = 9 with an ammonium hydroxide solution, added to a sodium sulfide solution (8 mmol of sodium sulfide nonahydrate dissolved in 10 mL of distilled water), and stirred for 1 h. The solution was transferred into a Teflon-lined stainless-steel autoclave (100 mL) and heat-treated in a muffle (Thermoconcept KLS 10/12, Mérignac, France) for 10 h at 140 °C. The product was washed with water and ethanol and centrifuged (Remi Elektrotechnik LDT., NEYA-8 centrifuge, Mumbai, India) at 6000 rpm for 10 min. The final product was separated from the liquid phase and dried in air overnight.

#### 2.2.2. Self-Standing Anodes

The solution to be electro-spun was prepared by dispersing 10% and 30 wt% ZnS–GO active material into a 8 wt% PAN in N,N-dimethylacetamide (10%ZnS–GO/CNF and 30%ZnS–GO/CNF samples) [31]. Hereafter we report details of the synthesis.

The ZnS–GO powder was ball-milled at 100 rpm for two cycles (20 min each), then 10 wt% of ZnS–GO (0.188 g) or 30 wt% of ZnS–GO (0.564 g) was added to 25 mL of N, N-dimethylacetamide, and the suspension was sonicated for 1 h, after which 1.88 g PAN was added and the suspension was stirred overnight at 60 °C. The obtained dispersion was electro-spun using a EF050—Starter Kit Electrospinning of SKE Research Equipment (C/O Leonardino S.r.l, Bollate, MI, Italy). The following conditions were selected to perform each deposition: 9 mL dispersion, 3.5 mL/h flow, 16 Gauge needle, applied voltage 14 kV, needle–collector distance 18 cm, deposition time 2.5 h. Finally, a homemade humidity sensor included box was built to control the humidity: a value lower than 20% was detected during each deposition.

**Figure 1:**
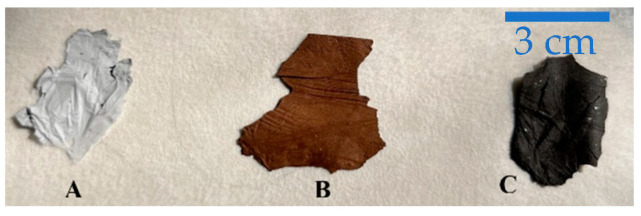
A portion of 10%ZnS–GO/CNF nanofibers after each heat treatment (**A**) post spinning, (**B**) post stabilization, (**C**) post carbonization.

The obtained fibers were removed from the support (aluminum foil) and stabilized in air for 30 min at 100 °C, 30 min at 200 °C, and finally 2 h at 260 °C (heating ramp: 5 °C min^−1^). The fibers were further heat-treated in a tubular furnace (Carbolite) at 750 °C for 2 h (heating ramp: 10 °C min^−1^) in nitrogen atmosphere for the carbonization process. Figure 1 shows a portion of the 10%ZnS–GO/CNF sample after each thermal treatment.

A sample of pure CNFs was also prepared for comparison. The same synthesis procedure was applied by omitting the addition of the active material to the 8 wt% PAN in DMAc solution.

#### 2.2.3. Tape-Casting Anode

The ZnS–GO active material was ball-milled at 100 rpm for two cycles (20 min each). To prepare the slurry, 70 wt% active material, 20 wt% Super P carbon and 10 wt% CMC binder were dispersed in distilled water and stirred for 2 h. The slurry was tape-casted (Doctor Blade coating technique) on copper foils and dried at 70 °C for 3 h.

### 2.3. Cell Assembly

The Swagelok-type cells were assembled in an Argon-filled dry box (M. Braun H_2_O < 0.1 ppm O_2_ < 0.1 ppm). NaClO_4_ 1M in EC:DEC (1:1) and 5 wt% FEC was used as electrolyte, and sodium foil as the counter-electrode.

### 2.4. Characterization Techniques

The samples were characterized by X-ray powder diffraction technique (XRPD). A Bruker D5005 diffractometer (Bruker, Karlsruhe, Germany) equipped with a Cu Kα radiation tube (40 kV, 40 mA), curved graphite monochromator on the diffracted beam and scintillation detector was used. The patterns were collected in the 17–80° 2-Theta range, step size 0.03° and counting time of 16 s/step. TOPAS 3.0 software (Bruker AXS, Karlsruhe, Germany) was used to apply the Rietveld structural refinement to the ZnS–GO and ZnS–GO/CNFs samples.

SEM micrographs of the synthesized samples were collected by a Zeiss EVO MA10 (Carl Zeiss, Oberkochen, Germany) microscope, equipped with an energy dispersive detector for the EDS analysis, on gold-sputtered samples (20 kV, secondary electron images, working distance 8.5 mm).

TEM images were collected on a JEOL JEM-1200EXIII equipped with TEM CCD camera Mega View III transmission electron microscope. The samples were dispersed in water; a drop of about 0.7 μL was deposited on the Ni grid and dried.

Thermogravimetric data were collected with a TGA Q5000 IR apparatus interfaced with a TA 5000 data station (TA Instruments, Newcastle, DE, USA) in the 25–750 °C temperature range in air (heating rate: 10 C min^−1^). This technique is used to evaluate the weight percentage of active material (ZnS–GO) loaded in the ZnS–GO/CNF samples.

Raman measurements were performed at room temperature using an automated and integrated confocal microRaman spectrometer, XploRA Plus HORIBA Scientific, equipped with an Olympus microscope BX43. Laser red light at 638 nm was used as excitation, tuning the 90 mW incident power by a set of neutral filters with different optical densities. The spectrometer is equipped with a motorized xy stage on which the investigated samples are positioned. Spectral resolution is about 2 cm^−1^. An Open Electrode CCD camera, with a multistage Peltier air-cooling system, is used as detector. The measurements were performed using a 50× magnification objective, with a spatial resolution of the order of 4 microns. The spectra were acquired with a mean integration time of about 20 s and a number of accumulations equal to 10. All the reported data are obtained as the average spectrum, sampling the materials in several different regions.

The electrochemical properties of the materials were investigated at ambient temperature by means of cyclic voltammetry (CV) and galvanostatic charge/discharge cycles using a Swagelok cell. The CV was performed with an Autolab PGSTAT30 potentiostat (Eco Chemie, Utrecht, The Netherlands) in the 0.01–3 V potential range, and the data were processed with GPES V4.9 software.

Galvanostatic charge/discharge cycles were obtained with a Neware-4000BTS Battery Testing System (Hong Kong, China) at different current rates in the 0.01–3 V potential range.

## 3. Results and Discussion

In the present work, the structure, morphology, and composition of the ZnS–GO, 10%ZnS–GO/CNF and 30%ZnS–GO/CNF samples were evaluated, and the electrochemical performance of the self-standing anodes was investigated and compared to the tape-casted example.

### 3.1. ZnS–GO and ZnS–GO/CNF Characterization

**Figure 2:**
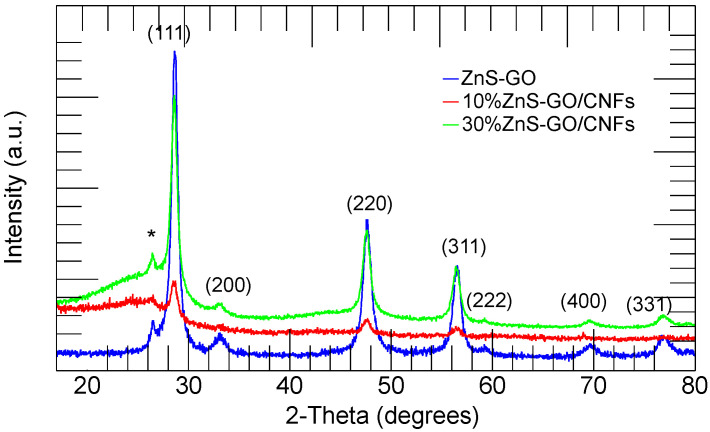
XRPD patterns of the ZnS–GO (blue), 10%ZnS–GO/CNF (red) and 30%ZnS–GO/CNF (green) samples. (002) plane of graphitic carbon (*) and Miller indices of the sphalerite phase are also shown.

The XRPD patterns of ZnS–GO, 10%ZnS–GO/CNF and 30%ZnS–GO/CNF samples are shown in Figure 2. The ZnS–GO pattern compares fairly to those reported in the literature [18]. The sample displays the peaks of ZnS in the sphalerite crystal structure (JCPDS: 05-0566): the peaks at 2-Theta values of 28.6, 33.1, 47.5, 56.3, 59.1, 69.5 and 76.8° are attributed to the (1 1 1), (2 0 0), (2 2 0), (3 1 1), (2 2 2), (4 0 0) and (3 3 1) planes, respectively. The peak detected at about 26.5° (2-Theta) is attributed to the (0 0 2) plane of carbon [18,23,44], and confirms the GO reduction to graphitized carbon.

The XRPD patterns of the ZnS–GO/CNF and the ZnS–GO samples are comparable: the ZnS–GO/CNF samples display the sphalerite phase and graphitized carbon peaks. In addition, a broad band centered at about 25° (2–Theta) is observed and attributed to the CNFs’ amorphous component. More intense peaks of the sphalerite phase are detected in the 30%ZnS–GO/CNF sample compared to the 10%ZnS–GO/CNF. This evidence confirms (i) the electrospinning process does not cause structural changes to the active material; (ii) a higher amount of active material is loaded in the 30%ZnS–GO/CNF sample.

The Rietveld refinement was applied to evaluate the lattice parameters and the crystallite size of the ZnS phase in each sample. The experimental and calculated patterns are shown in Figure S1, and the refined parameters are reported in Table S1. The ZnS–GO and ZnS–GO/CNF samples display comparable lattice parameters and a crystallite size of about 12 nm, in fair agreement with the literature values [18]. The value is also confirmed by applying the Scherrer equation to the (1 1 1), (2 0 0) and (3 1 1) reflections: the averaged values are 10, 11 and 12 nm for the ZnS–GO, 10%ZnS–GO/CNF and 30%ZnS–GO/CNF samples, respectively.

**Figure 3:**
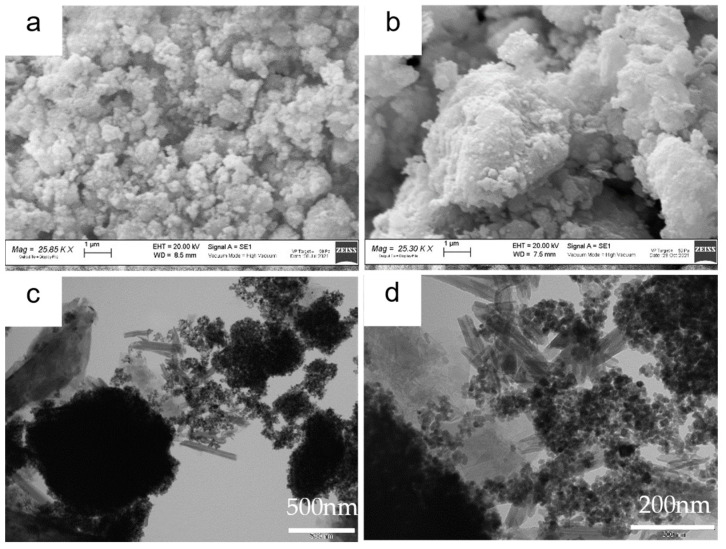
ZnS–GO powder: (**a**) and (**b**) SEM images at 25 kX, (**c**) and (**d**) TEM images at 50 kX and 150 kX, respectively.

The SEM images of the ZnS–GO sample are shown in Figure 3a,b. Agglomerates of variable dimensions, ranging between a few hundred nanometers and 10 μm, are detected. Figure 3b evidences that the agglomerates consist of stacked sheets covered by nanometric rounded particles. Indeed, the TEM images shown in Figure 3c,d confirms the presence of (i) nanoparticles of about 10–20 nm diameter, comparable to that evaluated by the Rietveld refinement for the ZnS sphalerite phase (Table S1) and (ii) cracked sheets. The results suggest that the sample consists of ZnS nanoparticles and graphene sheets. The cracked graphene foils are desirable, as they provide an intimate contact with the active material and a homogeneous dispersion of the composite in the slurry or in the solution for electrospinning.

**Figure 4:**
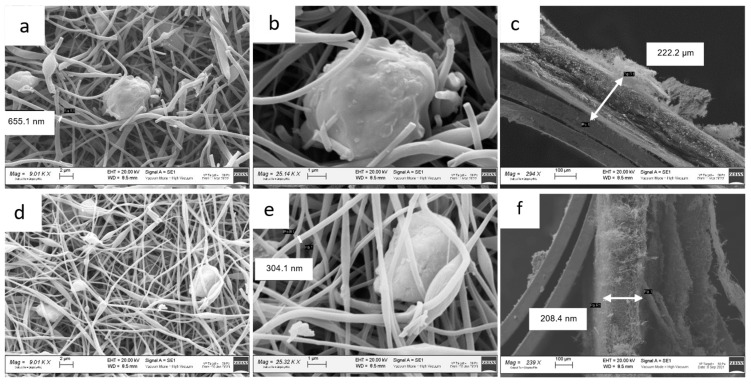
SEM images of 10%ZnS–GO/CNF sample: (**a**) and (**b**) at 9 and 25 kX, respectively, and (**c**) cross-section. SEM images of 30%ZnS–GO/CNF sample: (**d**) and (**e**) at 9 and 25 kX, respectively, and (**f**) cross-section.

Figure 4 shows the SEM images of the ZnS–GO/CNF samples after the carbonization process, ready to be used as self-standing anodes.

In Figure 4a,b, the images of the 10%ZnS–GO/CNF sample are shown, and Figure 4c displays its cross-section. The carbon nanofibers are clearly observed: they display a variable diameter that reaches values of about 650 nm. In the same figure, rounded agglomerates of variable size, both dispersed between the CNFs (Figure 4a,b) and embedded into them (Figure 4a, on the left), are detected and attributed to the active material loaded in the carbon nanofibers. The 10%ZnS–GO/CNF displays a sheet thickness of about 222 μm (Figure 4c).

In Figure 4d,e the images of the 30%ZnS–GO/CNF sample are shown, and Figure 4f displays the cross-section. Carbon nanofibers with an average diameter of about 300 nm are clearly observed. As for 10%ZnSGO/CNF, in the 30%ZnS–GO/CNF sample agglomerates of variable size are detected and are present at a higher amount, due to the higher quantity of ZnS–GO loaded. The sheet thickness is 208 µm, comparable to the value observed in the 10%ZnS–GO/CNF sample.

**Figure 5:**
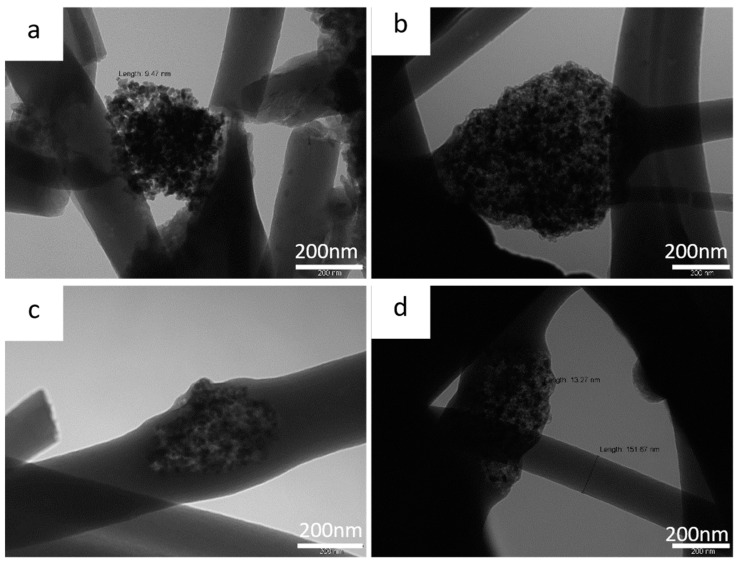
TEM images of (**a**) and (**b**) 10%ZnS–GO/CNF sample at 100 kX and (**c**) and (**d**) 30%ZnS–GO/CNF sample at 100 kX.

Figure 5 shows the TEM images of the ZnS–GO/CNF samples after the carbonization process.

In the 10%ZnS–GO/CNF sample (Figure 5a,b), the ZnS–GO agglomerates are clearly observed, both between nanofibers and connecting them, and within the nanofibers. Each agglomerate consists of nanometric particles of about 10 nm, while the CNF’s diameter is about 200 nm. The 30%ZnS–GO/CNF sample shows similar features. ZnS–GO nanoparticles of 13 nm are present, both between and embedded into nanofibers, and the CNF’s diameter is about 150 nm.

**Figure 6:**
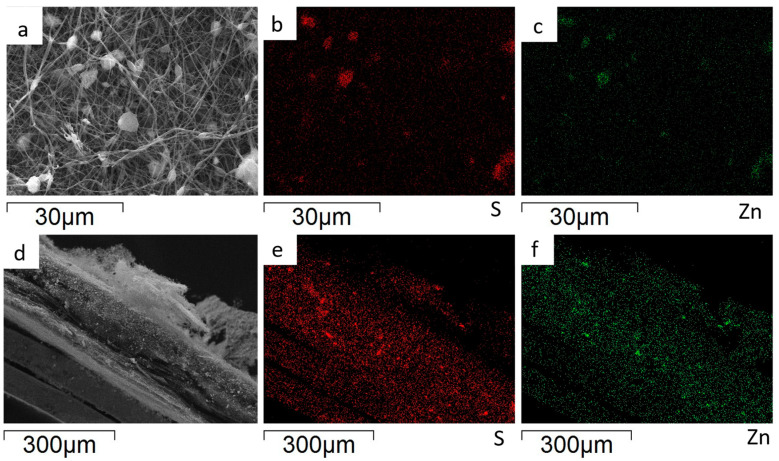
10%ZnS–GO/CNF anode: sample surface portion (**a**) and distribution maps of S (**b**) and Zn (**c**); cross-section portion (**d**) and distribution maps of S (**e**) and Zn (**f**).

The EDS analysis is applied to evaluate the ZnS agglomerate’s distribution on the surface and within the bulk of the ZnS–GO/CNF sheets. The Zn and S distribution maps of the 10%ZnS–GO/CNF sample are shown in Figure 6. The images taken on the sample surface (Figure 6a–c) confirm that the aggregates detected between and within the CNFs are ZnS particles. The cross-section images (Figure 6d–f) demonstrate that the ZnS–GO active material is homogeneously distributed along the sheet thickness, and no concentration gradients are observed. This feature is crucial for good electrochemical performance, and is seldom obtained when the active material is loaded by different synthetic approaches, such as dip-and drop-coating.

**Figure 7:**
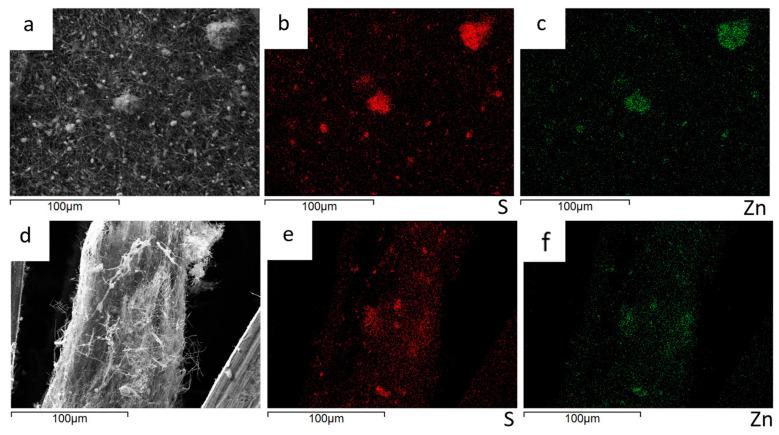
30%ZnS–GO/CNF anode: sample surface portion (**a**) and distribution maps of S (**b**) and Zn (**c**); cross-section portion (**d**) and distribution maps of S (**e**) and Zn (**f**).

The Zn and S distribution maps of the 30%ZnS–GO/CNF sample are shown in Figure 7. As for the 10%ZnS–GO/CNF sample, ZnS–GO aggregates are observed between and within the carbon nanofibers (Figure 7a–c) and the cross-section images (Figure 7d–f) confirm a homogeneous distribution of the active material along the sheet thickness.

As shown in Figure 6a and Figure 7a, ZnS graphene/CNF composites are indeed very segregated and the particle size distribution is also very broad. This finding is confirmed by the particle size distribution: we obtained 3.2(1.2) and 4.3(1.2) μm for the 10%ZnS–GO/CNF and 30%ZnS–GO/CNF samples, respectively. The aggregate’s size is not homogeneous and does not depend on the ZnS–GO amount loaded onto the CNFs.

The thermogravimetric analysis was carried out to evaluate the weight percentage of ZnS–GO loaded within the carbon nanofibers, to be compared to the amount used for the synthesis (10 and 30 wt%).

**Figure 8:**
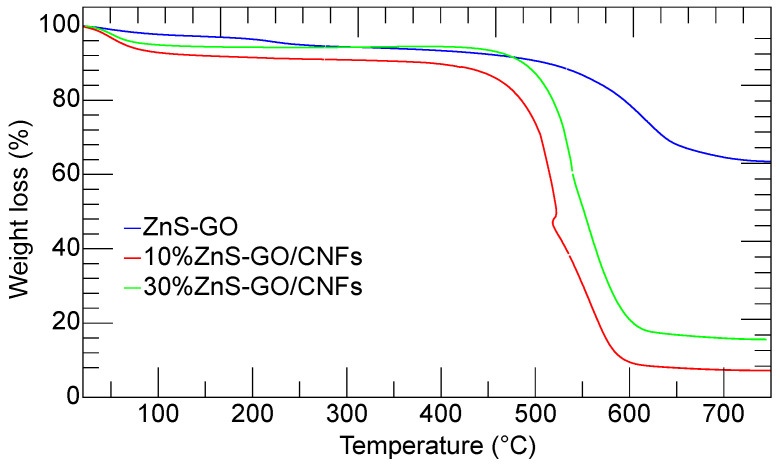
Thermogravimetric curves of ZnS–GO powder (blue), 10%ZnSGO/CNF sheet (red) and 30%ZnS–GO/CNF sheet (green).

The thermogravimetric curves of ZnS–GO, 10%ZnS–GO/CNF and 30%ZnS–GO/CNF samples are shown in Figure 8.

The ZnS–GO sample (blue line) shows two subsequent weight losses of 2.25 wt% and 35.33 wt% at 150 °C and 650 °C, respectively. As reported by Zhang et al. [18], the first loss is due to the release of water molecules. The second occurs in the 250–650 °C temperature range and is attributed to the ZnS and C oxidation in air according to Equations (1) and (2):

(1) $$ZnS+\frac{3}{2}O_{2}\;\to ZnO+SO_{2}$$

(2) $$C+O_{2}\;\to CO_{2}\;$$

At temperature higher than 650 °C, the sample weight is constant and attributed to the ZnO. From the ZnS–GO TG curve, a residual mass of 62.42 wt% is detected, and the calculated content of ZnS in the sample is about 75 wt%.

As in the case of ZnS–GO sample, the 10%ZnS–GO/CNF (red) and 30%ZnS–GO/CNF (green) TG curves both show weight losses. Again, the first at about 100 °C is due to the release of the water molecules. The latter occurs in the 250 °C–650 °C temperature range, and is attributed to carbonaceous component combustion (Equation (2)) ZnS oxidation (Equation (1)). As expected, the second mass loss in the ZnS–GO/CNFs samples is higher than in the ZnS–GO, as the combustion also involves the carbon nanofibers. The 10%ZnS–GO/CNF sample gives a residual mass of 7.15 wt% at 650 °C, due to the formation of ZnO. The calculated ZnS amount is 8.5 wt%, and the ZnS–GO amount is 11.3 wt% (the ZnS–GO powder contains 75% ZnS, as evaluated by TG data). The value matches that used in the synthesis (10 wt% of ZnS–GO). The 30%ZnS–GO/CNF sample gives a residual mass of 15.6 wt% at 650 °C; the calculated ZnS amount is 18.7 wt%, and the ZnS–GO amount is 24.9 wt%. This value compares fairly to that of the synthesis (30 wt%).

Raman spectroscopy provided information about these multicomponent materials. In particular, the technique allowed analysis of the structural changes of their carbonaceous parts at different preparation stages and comparison of the order degree of samples with different amounts of graphitic component [18].

In Figure S2, the room temperature Raman spectra are reported for the 30%ZnS–GO/CNF sample, as prepared after electrospinning, after stabilization and post-carbonization.

At the first two stages, the Raman yield is overwhelmed by a very broad and structureless signal, probably associated with fluorescence. Nevertheless, when the data are processed by subtracting a structureless background, a weak signal appears for the as-prepared sample in the region between 300–500 cm^−1^, where the vibrations of sphalerite ZnS structure should be active [45]. This Raman activity is accompanied for the stabilized sample by the appearance of the well-known Raman structures associated with graphene [46]. It is well known that the order/disorder and/or the crystalline quality of the carbonaceous materials are very well determined in Raman spectroscopy by monitoring the ratio between the integrated intensities of the G and D bands [47].

The G band at ~1580 cm^−1^ corresponds to the tangential C-C stretching vibration and is associated with the ordered sp^2^ hybridized carbon network. The peak at ~1330 cm^−1^, which is related to local defects that originate from structural imperfections, is referred to as the defect mode D-band, involving phonon emission, with the scattering of an electron by the disordered sp^3^ hybridized carbon network.

**Figure 9:**
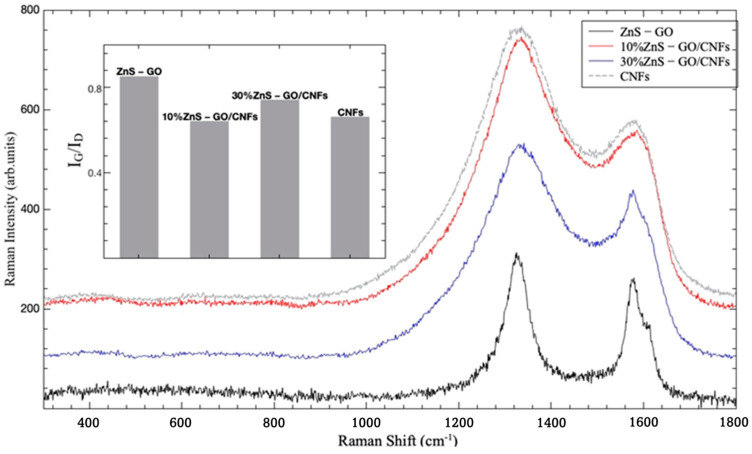
Room temperature Raman spectra for ZnS–GO (black line), 10%ZnS–GO/CNFs (red line) and 30%ZnS–GO/CNFs (blue line), together with the Raman spectrum for starting CNFs (gray line). In the inset, the intensity ratio (I_G_/I_D_) is reported for the same samples.

This strategy has thus been applied to the different carbonized materials. The results are shown in Figure 9, where the spectra for ZnS–GO, 10%ZnS–GO/CNFs and 30%ZnS–GO/CNFs are reported, together with the Raman spectrum for CNFs. One can appreciate the changes in line shapes and intensity ratios between G and D bands. Less significative are the very small changes in the peak energies. In the inset, the intensity ratio parameter (I_G_/I_D_) is reported for the four considered samples. The values for this parameter have been derived by best-fitting procedures in the range 1000–1800 cm^−1^ using a sum of Lorentzian curves as fitting functions, as shown in Figure S3 for the ZnS–GO sample and according to [48,49].

The higher value (0.85) is obtained for ZnS/GO composite, indicating a good crystalline order of the matrix. This is consistent with the presence of the graphene sheets embedding the ZnS nanoparticles and obtained by the graphene oxide reduction. When this matrix is added to CNFs, the I_G_/I_D_ value decreases and a net broadening of the line shape is observed. The lowest I_G_/I_D_ value (0.64) is obtained for 10%ZnS–GO/CNFs according to the lowest amount of the ordered carbon matrix; in this case, the value is practically equal to that obtained for pure CNFs. Increasing the amount of the ZnS–GO part leads to an increase of I_G_/I_D_ value (0.74) in any case lower than that for ZnS–GO.

These results evidence that the ordered carbon component present in the ZnS–GO/CNF samples is mainly related to the graphene embedding the ZnS nanoparticles. Indeed, the fitted peak position of the (0 0 2) reflection of carbon (see Figure S1) is very comparable for the three samples, and a d_002_ interplanar distance of 3.36 Å is calculated, independent of the presence of the CNF component and its amount.

### 3.2. ZnSGO and ZnS–GO/CNFs Electrochemical Characterization

**Figure 10:**
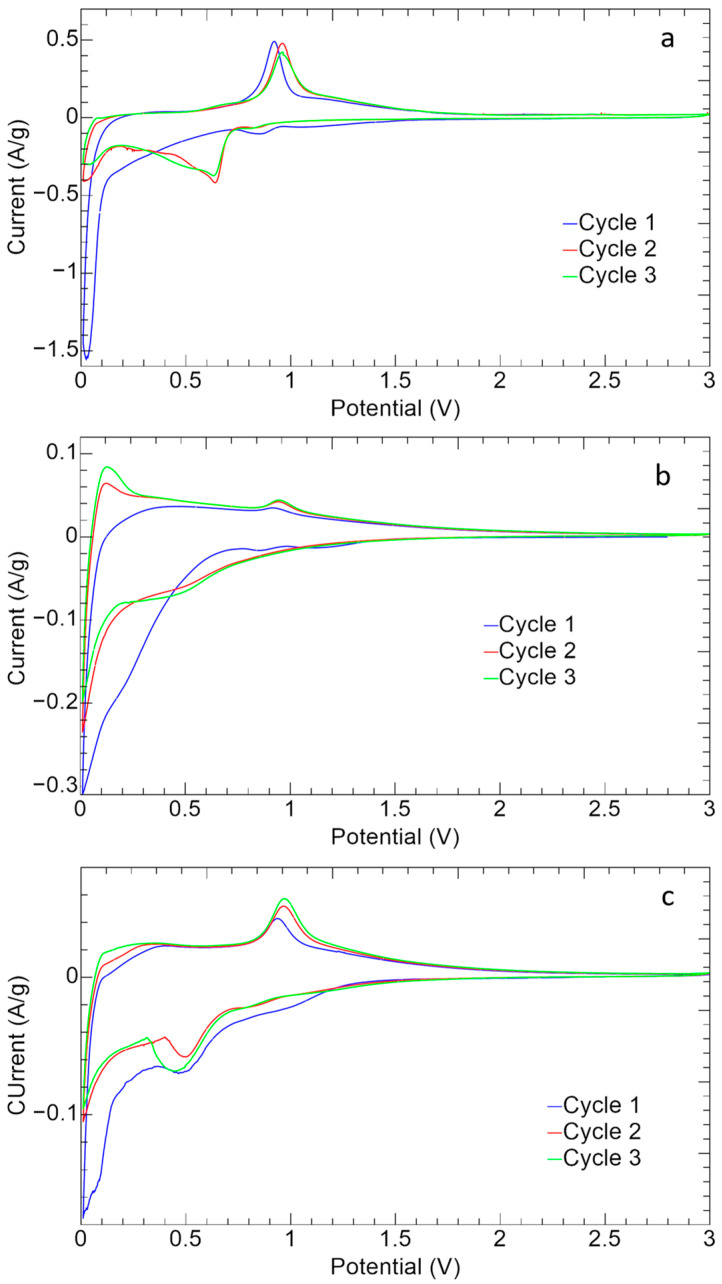
Cyclic voltammetry of (**a**) ZnS–GO, (**b**)10%ZnS–GO/CNF and (**c**) 30%ZnS–GO/CNF samples.

The cyclic voltammetry curves of the ZnS–GO, 10%ZnS–GO/CNF and 30%ZnS–GO/CNF samples are shown in Figure 10.

All the samples show the typical redox peaks attributed to the ZnS reversible redox reaction [18,50]:

(3) $$nS+xNa^{+}+xe^{-}\;\to Na_{x}S+Zn$$

(4) $$Na_{x}S+\left(2-x\right)Na^{+}+\left(2-x\right)e^{-}\;\to Na_{2}S$$

(5) $$n+Na_{2}S\;\to 2Na+ZnS$$

The reduction (Equations (3) and (4)) and oxidation (Equation (5)) peaks are detected at 0.5 V–0.7 V and at about 1.0 V, respectively. During the cathodic cycle, the insertion of Na^+^ and the conversion of ZnS to metallic Zn occurs (Equations (3) and (4)) [18], while in the anodic process the conversion of Zn to ZnS and the extraction of Na^+^ takes place (Equation (5)) [18]. The first cycle presents, in the cathodic region, the broad peak between 0.1 V–0.5 V attributed to the SEI formation, caused by the intercalation of Na^+^ and the structure settling [18].

In the case of the ZnS–GO sample (Figure 10a), the anodic and cathodic peaks display a current intensity higher than 0.5 A g^−1^, and quite high ΔV values of about 0.3 V, which highlight polarization phenomena. While the anodic peaks are detected at 1 V, the cathodic peak at 0.9 V moves to 0.5 V–0.7 V after the first cycle and the formation of SEI. Finally the peaks are not perfectly overlapped, confirming that the redox reversibility is not strong.

In the 10%ZnS/CNF CV (Figure 10b), the redox peaks are broader and display lower current intensities than the ZnS–GO (Figure 10a). It should be underlined that, in the self-standing anode, the active material amount is only 11.3 wt% vs. 70% in the slurry ZnS–GO electrode. Noteworthy, for the 10%ZnS–GO/CNF sample the redox peaks are overlapped, suggesting a good reversibility of the electrochemical process. In the anodic region, a redox peak is also detected at about 0.1 V. This can be due to the CNF component [37,51], as the sample contains about 90 wt% of carbon nanofibers.

In the CV curve of the 30%ZnS–GO/CNF (Figure 10c), the redox peaks display currents of 0.05 A g^−1^ in charge and −0.08 A/g in discharge. These values are lower than the slurry electrode but higher than the 10%ZnS–GO/CNF. This is explained by the ZnS–GO powder amount in the 30%ZnS–GO/CNF: lower than the 70% of slurry, but higher than the 11.3% in the other self-standing electrode. In this case also it is possible to see the formation of SEI in the first cycle.

**Figure 11:**
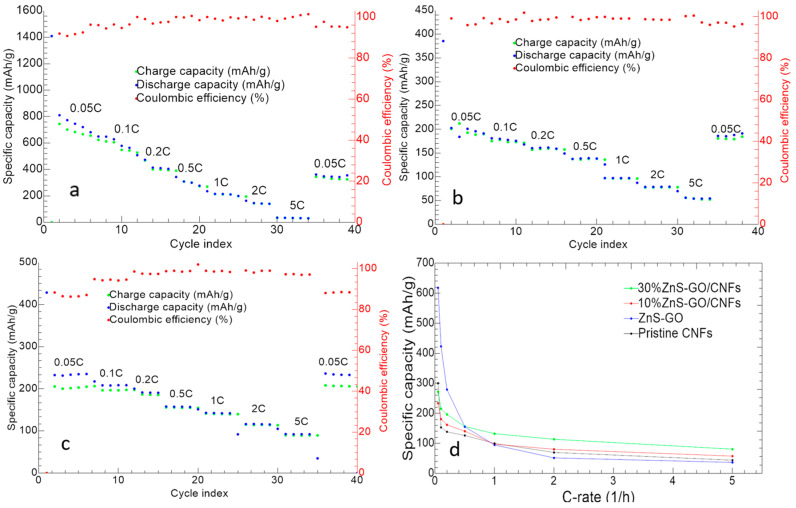
Charge/discharge cycles at different C-rates of (**a**) ZnS–GO, (**b**) 10%ZnS–GO/CNF and (**c**) 30%ZnS–GO/CNF samples. (**d**) Samples’ capacity vs. C-rates. Charge (green), discharge (blue) and coulombic efficiency (red).

The charge/discharge cycles at different C-rates of ZnS–GO, 10%ZnS–GO/CNF and 30%ZnS–GO–CNF samples are reported in Figure 11.

The ZnS–GO (Figure 11a) displays an initial discharge capacity of 1409 mAh g^−1^ and Initial Coulombic Efficiency (ICE) of 58.89%. CE increases to 91.93% in the second cycle. The initial capacity loss is attributed both to the SEI formation and Na^+^ trapping. Averaged capacities of 671.93, 423.14, 279.27, 155.42, 94.97, 51.72 and 36.76 mAh g^−1^ are reached at 0.05 C, 0.1 C, 0.2 C, 0.5 C, 1 C, 2 C and 5 C, respectively. After the first cycle, a progressive capacity loss is observed for each C-rate, until the cell reaches good stability and a high overlapping of charge and discharge capacity, suggesting a reversibility of the electrochemical process. The good electrochemical performance is also confirmed by the value of the coulombic efficiency at 98%. At the end of the analysis, the capacity at 0.05 C is 43.89% of the initial one after SEI formation.

The discharge capacity in the first cycle of the 10%ZnS–GO/CNF sample (Figure 11b) is 385.3 mAh g^−1^. The ICE is 52.10%, and increases to 99.16% in the second cycle. Averaged specific capacities of 233.79, 181.06, 162.01, 140.57, 97.42, 80.55, and 57.68 mAh g^−1^ are obtained at 0.05 C, 0.1 C, 0.2 C, 0.5 C, 1 C, 2 C and 5 C, respectively. By switching again to 0.05 C, a good capacity recovery is obtained, corresponding to 94.41% of initial capacity after SEI formation.

The 30%ZnS–GO/CNF self-standing electrode (Figure 11c) gives an initial discharge capacity of 428.7 mAh g^−1^. The ICE value of 57.95% increases to 88.34% in the second cycle. Averaged specific capacities of 271.89, 215.6, 196.38, 156.25, 132.28, 113.83 and 80.80 mAh g^−1^ are obtained at 0.05 C, 0.1 C, 0.2 C, 0.5 C, 1 C, 2 C and 5 C, respectively. At the end of the measurement, capacity at 0.05 C is totally recovered.

Both self-standing anodes display an increased stability compared to the slurry electrode, as a lower capacity loss is detected by increasing the C-rate. Noteworthy, the electro-spun electrodes display an improved capacity recovery compared to the ZnS–GO anode obtained by tape-casting.

In Figure 11d, the specific capacity of the three electrodes as a function of C-rate is shown and compared to the electrochemical activity of the pristine CNF anode. The capacity value is the average specific capacity at each C-rate. For all anodes, the specific capacity gradually decreases as C-rate increases, but for the self-standing electrodes the capacity decay is not so steep as for the slurry anode. Moreover, both the 10%ZnS–GO/CNF and 30%ZnS–GO/CNF samples display fair capacity values at high C-rates: 69.93 and 92.59 mAh g^−1^ at 5 C, respectively, vs. 50 mAh g^−1^ for the ZnS–GO anode. Notably, the electrochemical activity of the pristine CNFs self-standing electrode stands between the slurry and self-standing anodes. At low C-rate, the pristine CNF anode shows lower capacity than slurry, 10%ZnS–GO/CNF and 30%ZnS–GO/CNF electrodes, while at C-rate higher than 1 C. the pristine CNFs displays capacity values higher than slurry but lower than both 10%ZnS–GO/CNF and 30%ZnS–GO/CNF anodes. At 1 C, the pristine CNFs, 10%ZnS–GO/CNF and slurry electrodes show comparable electrochemical performance.

The obtained charge/discharge results suggest that the self-standing anodes display a lower value of specific capacity at low C-rates, due to the lower amount of active material in the electrode, but seem really promising at high C-rates.

**Figure 12:**
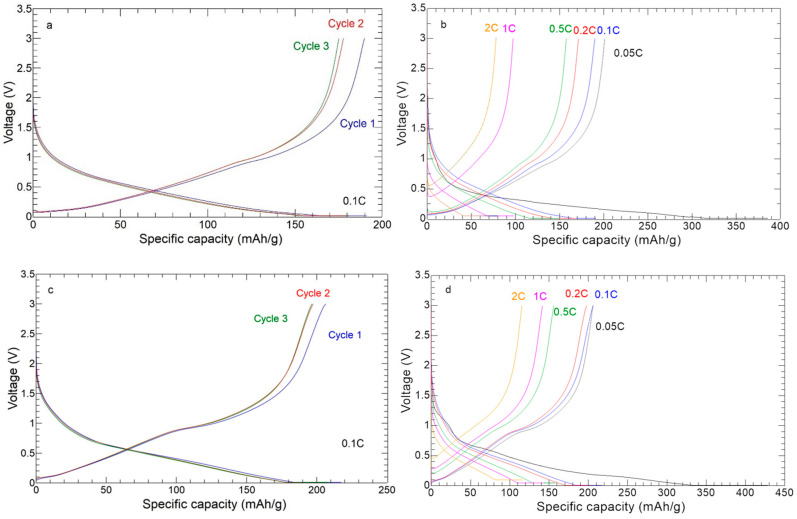
Charge/discharge curves: (**a**) three cycles at 0.1 C of 10%ZnS–GO/CNF; (**b**) one cycle at each C-rate of 10%ZnS–GO/CNF; (**c**) three cycles at 0.1 C of 30%ZnS–GO/CNF; (**d**) one cycle at each C-rate of 30%ZnS–GO/CNF.

The galvanostatic charge–discharge profiles of the self-standing anodes are shown in Figure 12. The GCD profiles at 0.1 C (Figure 12a,c) agree with the CV results. The high overlap of the second and third cycle curves of the 30%ZnS–GO/CNF sample suggests a satisfactory reversibility of charge and discharge processes. The curve’s shape compares well to ZnS anodes reported in the literature [22,23]. The voltage plateau at about 1 V is more evident in the 30%ZnS–GO/CNF sample, containing a higher amount of active material (24.9 vs. 11.3 wt%). The charge–discharge profiles at different C-rates (Figure 12b,d) confirm higher capacity at high C-rate in the 30%ZnS–GO/CNF sample.

**Figure 13:**
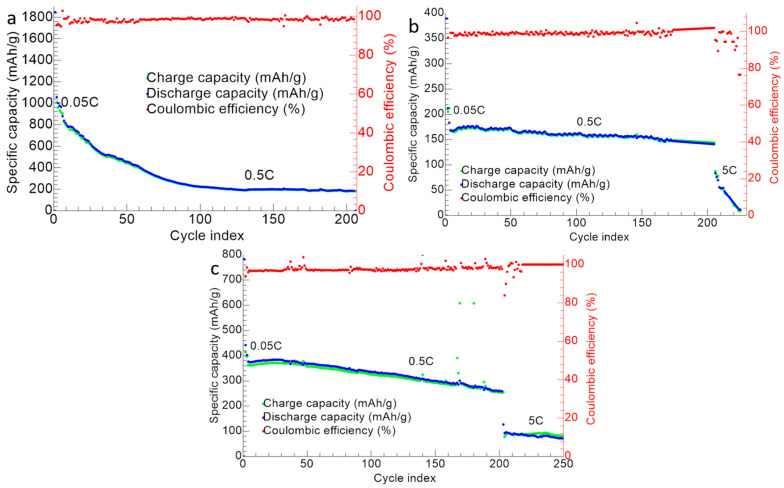
Cycling performance of (**a**) ZnS–GO, (**b**) 10%ZnS–GO/CNF and (**c**) 30%ZnS–GO/CNF samples. Charge (green), discharge (blue) and coulombic efficiency (red).

Figure 13 shows the cycling performance for the ZnS–GO, 10%ZnS–GO/CNF and 30%ZnS–GO/CNF samples. The cells were tested at 0.5 C for 200 cycles, and the ZnS–GO/CNF electrodes were also cycled at 5 C for 50 cycles.

In the case of ZnS–GO (Figure 13a), the initial capacity at 0.05 C is 1847 mAh g^−1^. In the following cycles at 0.5 C, a gradual capacity loss is detected, and the capacity reaches stable values (in the range 185–223 mAh g^−1^) only after the 100th cycle. The charge and discharge capacity values are overlapped and a coulombic efficiency ≥98% is obtained. The cell was also tested at 5 C after 200 cycles at 0.5 C, but this did not work. The capacity retention after 200 cycle is 21.20%.

For the 10%ZnS–GO/CNF sample (Figure 13b), the initial capacity is 389.6 mAh g^−1^ at 0.05 C. In the following cycles, the capacity decreases, but the cell already reaches a stable capacity value (about 150 mAh g^−1^) at the 13th cycle. The charge/discharge capacity values are overlapped and coulombic efficiencies ≥99% are obtained at 0.5 C. In this case, the cell could be tested at 5 C for 50 cycles, after 200 cycles at 0.5 C. In comparison to the slurry ZnS–GO electrode, the 10% self-standing anode reaches stable capacity values quicker and withstands cycling at high C-rate after 200 cycles at 0.5 C. The capacity retention after 200 cycle is 66.51%.

Finally, the 30%ZnS–GO/CNF sample (Figure 13c) has an initial capacity of 789 mAh g^−1^ at 0.05 C. In the following 200 cycles at 0.5 C, the cell displays better cyclability than the slurry, but worse than the 10% self-standing anode. Interestingly, the 30% self-standing electrode displays capacity values in the 255–400 mAh g^−1^ range for 200 cycles at 0.5 C. The charge and discharge capacities are overlapped and coulombic efficiencies ≥99% are reached. The electrode also displays good cycling performances at 5 C for other 50 cycles, showing a capacity of 80–90 mAh g^−1^, and the cell still works at the end of the cycling test. The capacity retention after 200 cycles is 69.57%.

We can compare the electrochemical results obtained at 0.5 C for the 30%ZnS–GO/CNF sample (ZnS: 24.9 wt%) to recent literature results for metal sulfide/CNF anodes synthesized by electrospinning and used for SIBs. Bell string-like hollow ZnS–CNF (ZnS: 50 wt%) displays a reversible capacity of 361.7 mAh g^−1^ at 0.2 A g^−1^ and 433.5 mAh g^−1^ after 50 cycles at 0.1 A g^−1^ [23]. Capacity values of about 510 mAh g^−1^ at 0.2 A g^−1^ and 499.9 mAh g^−1^ after 100 cycles at 0.1 A g^−1^ were reached by low crystallinity SnS encapsulated in CNT decorated and S-doped CNF anodes (SnS: 48.3 wt%) [52]. Finally, rGO-encapsulated MoS_2_/CNF electrodes (Sulphur: 20.9 wt%) display a capacity of 345.8 mAh g^−1^ at the 90th cycle at 0.1 A g^−1^ [53] and 3D-hierarchical MoS_2_-CNF nanostructures (MoS_2_: 63 wt%) retain a capacity of 438 mAh g^−1^ after 100 cycles at 0.1 A g^−1^ [54]. Our results with the 30%ZnS–GO/CNF sample seems appealing, if we take into consideration the lower amount of active material loaded onto CNFs (ZnS: 24.9 wt%).

The 30%ZnS–GO/CNF anode electrochemical performances suggest that the self-standing electrode investigated in this study is very promising in terms of improving the specific capacity at high C-rate and the lifespan of the cell. This goal is obtained thanks to the role played by both the graphene sheets embedding the ZnS nanoparticles and the CNFs. The ordered carbon component, suitable for increasing the poor electronic conductivity of ZnS, mainly resides in the graphene sheets coating the active material, as demonstrated by the Raman spectroscopy results. Instead, the carbon nanofibers properly buffer the huge volume changes during the sodiation/desodiation processes: this explains the improved cycling performances at high C-rate with respect to the conventional tape-casted anode. Notably, the improvement is obtained by using a lower amount of active material (24.9 wt% vs. 70 wt% of the tape-casted anode), and neither metal support nor binder is necessary. Finally, a feasible and simple two-step synthesis was used. In our study, we prepared ZnS–graphene composites and simply added them to the carbon precursor solution to be electro-spun, stabilized and carbonized, and we obtain the ZnS–graphene embedded in carbon nanofibers. This synthetic strategy may be extended to other active materials: in the first step, they can be properly modified, coated or embedded to improve their electrochemical performance, then a further upgrade can be reached simply by adding the composite to the carbon precursor solution employed for electrospinning.

## 4. Conclusions

In the present work, ZnS–graphene anode material was synthesized and loaded onto carbon nanofibers by an electrospinning technique. The self-standing and binder-free electrodes obtained were characterized. Their electrochemical performances in a sodium half-cell were tested and compared to the ZnS–graphene anode obtained by conventional tape-casting deposition on copper foil.

It was shown that ZnS–graphene composite was synthesized. ZnS nanoparticles (about 10 nm) crystallize in the sphalerite structure and are inserted onto graphene sheets by graphene oxide reduction during the hydrothermal synthesis process. The obtained composite was then loaded (11.3 and 24.9 wt%, as evaluated by TG analysis) onto CNFs: ZnS–GO agglomerates are detected between CNFs and embedded inside them, as demonstrated by SEM and TEM analysis. They are homogeneously distributed on the surface and along the thickness of the CNFs. The electrochemical tests demonstrate that both free-standing anodes display improved electrochemical performances in terms of specific capacity at C-rates higher them 1 C, compared to the conventional tape-casted anode (69.93 and 92.59 mAh g^−1^ at 5 C for 10%ZnS–GO/CNF and 30%ZnS–GO/CNF samples, respectively, vs. 50 mAh g^−1^ for ZnS–GO). The best cycling performances are obtained for the 30%ZnS–GO/CNF sample (effective loading of 24.9 wt% active material): this displays capacity values in the 255–400 mAh g^−1^ range for 200 cycles at 0.5 C and coulombic efficiencies higher than 99%, and capacities of 80–90 mAh g^−1^ for another 50 cycles at 5 C.

We recall that different approaches are reported in the literature to preparation of the active material–CNF composites by employing the electrospinning technique. They are mainly based on (i) dip- or drop-coating the active material precursors on electro-spun CNFs; (ii) mixing the active material and carbon precursors and electrospinning them. In both cases, the samples need thermal- and chemical-treatments to synthesize in situ the active material. In the present work, we demonstrate that improved electrochemical performances can be obtained by a simple and feasible approach: ex situ synthesis of the active material, alone or optimized (in our case, embedded into graphene sheets, the source of ordered carbon, as demonstrated by Raman spectroscopy results), and its addition to the carbon precursor solution to be electrospinning.

## Data Availability

The data presented in this study are available on request from the corresponding author.

## Supplementary Materials

The following supporting information can be downloaded at: https://www.mdpi.com/article/10.3390/nano13071160/s1, Figure S1: Rietveld refinement of the X-ray diffraction data of the ZnS–GO, 10%ZnS–GO/CNF and 30%ZnS–GO/CNF samples; Figure S2: Room temperature Raman spectra for the sample 30%ZnS–GO/CNF as obtained after electrospinning, after stabilization and post carbonization process; Figure S3: Result from best-fitting procedure performed in the range 1000–1800 cm^−1^ on the Raman data from Zn–GO sample; Table S1: Lattice parameters, crystallite size, weighted discrepancy factor and Goodness of Fit obtained by the Rietveld refinement of the diffraction data of the ZnS–GO, 10%ZnS–GO/CNF, 30%ZnS–GO/CNF samples.
