# Supplementary material for: ZnS–rGO/CNF Free-Standing Anodes for SIBs: Improved Electrochemical Performance at High C-Rate
Source: Nanomaterials (Basel). 2023 Mar 24;13(7):1160. doi: 10.3390/nano13071160 (PMC10097268; doi:10.3390/nano13071160)
Supplement: Supplementary file 1 [file nanomaterials-13-01160-s001.zip › nanomaterials-2271415-supplementary.pdf]

ZnS-rGO/CNF free-standing anodes for SIBs: improved electrochemical performance at high C-rate

Debora Maria Conti [1], Cristina Fusaro [1], Giovanna Bruni [1], Pietro Galinetto [2], Benedetta Albini [2], Chiara Milanese [1], Vittorio Berbenni [1], Doretta Capsoni [1,*]

[1] Department of Chemistry, Physical Chemistry Section & C.S.G.I. (Consorzio Interuniversitario per lo Sviluppo dei Sistemi a Grande Interfase), University of Pavia, 27100 Pavia, Italy; deboramaria.conti01@universitadipavia.it (D.M.C.); cristina.fusaro01@universitadipavia.it (C.F.); giovanna.bruni@unipv.it (G.B.); chiara.milanese@unipv.it (C.M.); vittorio.berbenni@unipv.it (V.B.)

[2] Department of Physics, University of Pavia, 27100 Pavia, Italy; pietro.galinetto@unipv.it (P.G.); benedetta.albini01@universitadipavia.it (B.A.)

* Correspondence: doretta.capsoni@unipv.it (D.C.) Tel.: +39-0382-987213

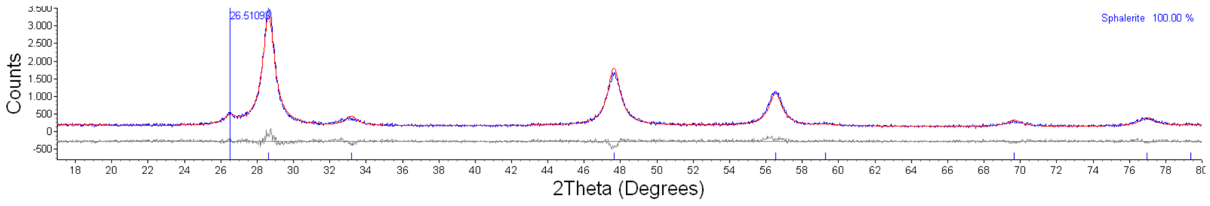

(a)

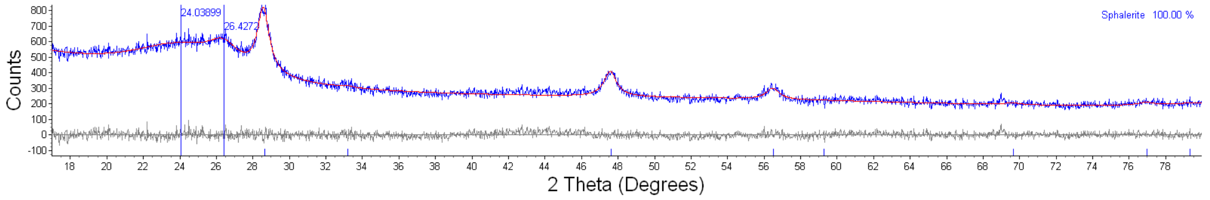

(b)

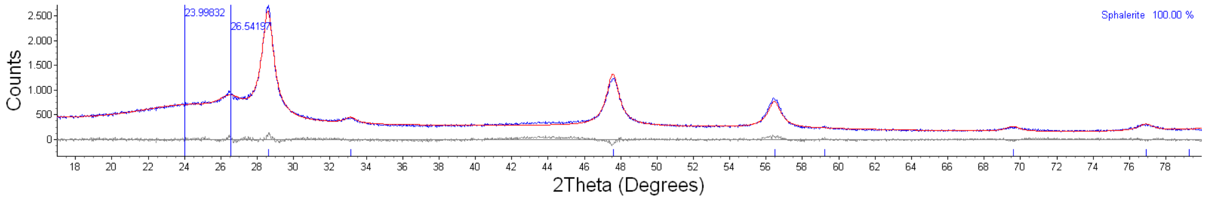

(c)

Figure S1. Rietveld refinement of the X-ray diffraction data of the a) ZnS-GO, b) 10%ZnS-GO/CNF and c) 30%ZnS-GO/CNF samples. Experimental pattern (blue line), calculated pattern (red line), difference curve (gray line). Peaks position of the sphalerite phase (bottom: blue bars). Peaks position of the (0 0 2) plane of graphene and of the amorphous carbon (blue vertical lines).

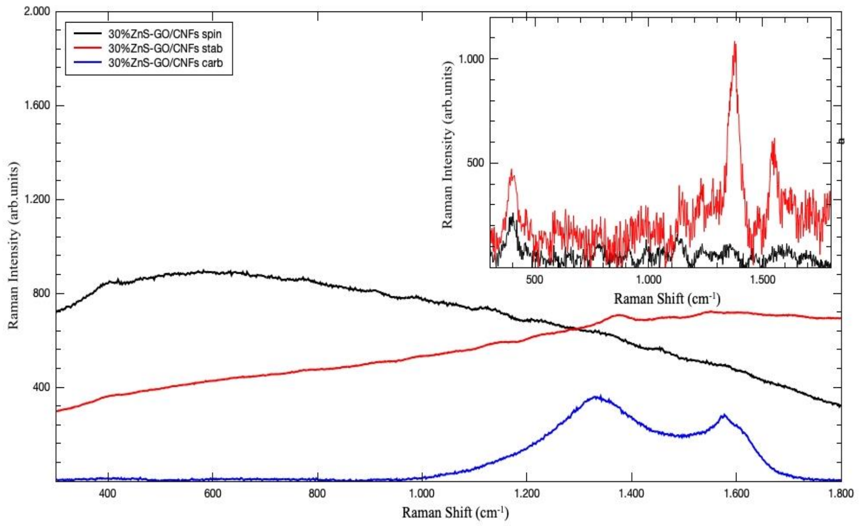

Figure S2. Room temperature Raman spectra for the sample 30%ZnS-GO/CNF as obtained after electrospinning (black line), after stabilization (red line) e post carbonization process (blue line). The inset shows the first two spectra as derived after baseline subtraction using a polynomial curve.

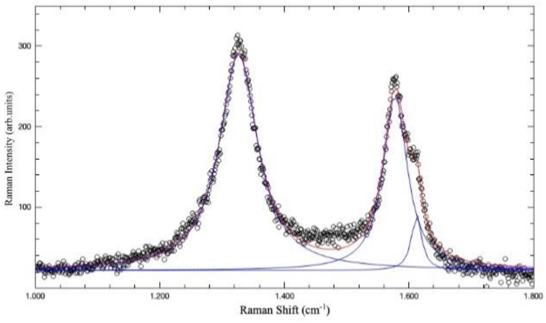

Figure S3. Result from best-fitting procedure (red line) performed in the range 1000-1800 cm^{-1} on the Raman data (empty circle) from Zn-GO sample using a sum of 3 lorentzian curves (blue lines). From the fitting parameters the values for (I_G/I_D) have been derived.

Table S1. Lattice parameters, crystallite size, weighted discrepancy factor and Goodness of Fit obtained by the Rietveld refinement of the diffraction data of the ZnS-GO, 10%ZnS-GO/CNF, 30%ZnS-GO/CNF samples.

**SAMPLE**	**ZnS-GO**	**10%ZnS-GO/CNF**	**30%ZnS-GO/CNF**
**a (Å)**	5.3947(7)	5.3961(32)	5.3986(7)
**Crystallite size (nm)**	11.8	11.7	11.7
**R_{wp}**	9.7	6.2	5.4
**GoF**	1.7	1.1	1.1
